# Happiness and arousal: framing happiness as arousing results in lower happiness ratings for older adults

**DOI:** 10.3389/fpsyg.2015.00706

**Published:** 2015-06-05

**Authors:** Par Bjalkebring, Daniel Västfjäll, Boo E. A. Johansson

**Affiliations:** ^1^Department of Psychology, University of Gothenburg, Gothenburg, Sweden; ^2^Department of Behavioral Sciences and Learning, Linkoping University, Linkoping, Sweden; ^3^Decision Research, Eugene, OR, USA

**Keywords:** happiness, age, arousal, age-differences, emotion

## Abstract

Older adults have been shown to describe their happiness as lower in arousal when compared to younger adults. In addition, older adults prefer low arousal positive emotions over high arousal positive emotions in their daily lives. We experimentally investigated whether or not changing a few words in the description of happiness could influence a person’s rating of their happiness. We randomly assigned 193 participants, aged 22–92 years, to one of three conditions (high arousal, low arousal, or control). In line with previous findings, we found that older participants rated their happiness lower when framed as high in arousal (i.e., ecstatic, to be bursting with positive emotions) and rated their happiness higher when framed as low in arousal (i.e., satisfied, to have a life filled with positive emotions). Younger adults remained uninfluenced by the manipulation. Our study demonstrates that arousal is essential to understanding ratings of happiness, and gives support to the notion that there are age differences in the preference for arousal.

## Introduction

How can happiness be measured in a reliable way? To ask “How happy are you?” is a straightforward question, and research suggests that this method has high validity. However, more elaborate ways to measure happiness have also been proposed (see Layard, 2011). During the last decades there has been an increasing interest in happiness and comparisons of happiness. Some examples are happiness comparisons based on country of residence, where Danes are among the happiest (Veenhoven, 1995; Diener et al., 2010); wealth, where wealthier people are shown to be happier than less wealthy people (Easterlin, 1995; Clark et al., 2008); and age, where younger people are shown to be happier than older people (Mroczek and Kolarz, 1998; Holahan et al., 2008). Does this mean that to be really happy one needs to be a young, rich Dane? The answer is probably no. When it comes to age-related differences in happiness, there are disparities in the findings, and the evidence that happiness changes as we age has been challenged (see Frijters and Beatton, 2012). Methodologically, the validity of all happiness comparisons rests on the assumption that “happiness” has the same meaning across all groups compared, and does not change throughout the lifespan.

Mogilner et al. (2011) investigated how people of different ages described their happiness in more than 12 million personal blogs, and they concluded that older adults described their happiness more often as satisfaction (low in arousal) and that younger adults described it more often as excitement (high in arousal). This is in line with Russell’s (2003) notion that emotions can vary in both valence (negative–positive) and arousal (low activation—high activation), and that high arousal has been argued to be connected to cognitive costs, psychological costs, and health costs in older adults (Williams et al., 1996; Bradley and Lang, 2000; Wurm et al., 2004). Kunzmann et al. (2013), showed that anger (high arousal) decreased with age, while sadness (low arousal) remained stable, and suggested that both positive and negative high arousal emotions will decline over the lifespan. Additionally, research has found that older adults rate their life as more positive, especially when it comes to low arousal positive affect (Kessler and Staudinger, 2009; English and Carstensen, 2014). It has also been shown that older adults have a clear preference for low arousal positive affect over high arousal positive affect when asked to value their experiences (Scheibe et al., 2013). In line with these findings, this study aims to experimentally investigate the impact of an arousal framing on happiness ratings.

Growing older is associated with decline in deliberative processes (Salthouse, 1991). It is less clear, however, how affective and emotional processing changes across the lifespan. Older adults appear to attend to and remember relatively more affective content compared to non-affective content (and sometimes positive content specifically), compared to younger adults (Mather and Carstensen, 2005). Positivity bias in older adults, both in terms of positivity enhancements and negativity reductions, has been demonstrated in various contexts. For example, older adults tend to prefer positive memories (Kennedy et al., 2004; Mather and Carstensen, 2005), pay more attention to positive information (Isaacowitz et al., 2006; Orgeta, 2011), and are more likely to prefer alternatives that are associated with positive affect (Mather and Johnson, 2000; Mather et al., 2004; Löckenhoff and Carstensen, 2007; Kim et al., 2008). In sum, previous studies have typically suggested that older adults prefer positive information when compared to younger adults. This positivity bias would suggest that older adults rate their happiness as higher than younger adults, however, longitudinal studies have found that age is associated with less happiness as well as greater decline in happiness (Mroczek and Kolarz, 1998; Holahan et al., 2008).

One explanation could be that affective preference is not only based on valence, but also based on arousal (Russell, 2003). Imagine a choice between talking to a person who will make you feel frustrated or to someone who will make you feel bored. If choices among affective states are based on a single valence dimension, one should expect indifference between these “equally” unpleasant affects. If, on the other hand, valence and arousal jointly determine attractiveness, we should expect that differences in activation would influence the preference for which person you would rather talk to. Preference for arousal may also be situational (Västfjäll and Gärling, 2006). In addition, it has been argued that preference for arousal varies over the lifespan, meaning that older adults are more prone to avoid high arousal emotions, when compared to younger adults (Carstensen, 2006; Peters et al., 2007). In a meta-analysis based on more than 100 studies, Pinquart (2001) showed that an age-related decline in emotional experiences is driven predominantly by high arousal emotions, irrespective of valence. It has also been suggested that part of the positivity bias might be attributed to “arousal avoidance” in older adults, as positive information is less arousing than negative information (Wurm et al., 2004).

In the present research we examined the influence of arousal by framing happiness as a low or high arousal state. Based on Russell’s (2003) notion that preference for affect can change when arousal changes, as well as previous research on age differences in arousal preference (Kessler and Staudinger, 2009; Mogilner et al., 2011; English and Carstensen, 2014), we expect that older adults will rate their happiness lower when it is framed as high in arousal (ecstatic), and higher when it is framed as low in arousal (satisfied).

## Materials and Methods

We advertised in a local newspaper to invite participants to a study of emotions and decision-making. Among 400 respondents, we randomly selected 250 who were invited to participate in a study specifically on well-being and emotions. A total of 193 participants (77% of the contacted sample) participated in the study. Age ranged from 22 to 92 years (*M* = 56 years, SD = 20 years; 37% men). The relatively high mean age is an effect of a higher response rate in older participants compared to younger participants. All 193 participants were used in all data analyses.

After completing a page on demographics, age, gender, education, income and occupation the participants turned the page and came to the main part of our study. Critical to our experiment, we randomly assigned participants to one of three arousal framing manipulations in which we, with a small change in wording, changed the arousal level in the description of happiness written on the top of the page. The definition was stated at the top of the page and the participants read this definition before performing the happiness ratings: (1) control manipulation, no definition was present, only “rate your happiness”; (2) low arousal framing manipulation, “happiness is to be satisfied, to have a life filled with positive emotions”; (3) high arousal framing manipulation, “happiness is to be ecstatic, to be bursting with positive emotions.” After reading this, the participants continued to rate their happiness at different time points. Participants were asked “All things considered, how happy are you right now?” In addition, we asked how happy they had been 10 years ago, 1 year ago, and yesterday, as well as how happy they thought they would be tomorrow, in 2 weeks, in 2 months, in 1 year, and in 10 years. Happiness was rated on a five-point Likert scale (1 = not at all happy, 5 = very happy). After answering these questions they turned the page and answered items about their emotional well-being, such as the SWLS (Diener et al., 1985). These additional emotion-related questions were not used in any of our analyses and were mainly added for face validity as the main part of the experiment was only two pages.

## Results

The participants were well educated (58% had a college or University degree), with a mean income of 40,000 dollars (SD = 11,500 dollars), and rated their subjective wellbeing as average (*M* = 4.79, SD = 1.33). Age was negatively related to education (*r* = 0.37, *p* < 0.001), but was not related to income (*r* = –0.07, *p* = 0.31) nor well-being (*r* = 0.10, *p* = 0.18). To insure that our randomization worked, we investigated if the three groups (low arousal, control and high arousal) differed in education [*F*(190,2) = 0.05, *p* = 0.949], income [*F*(189,2) = F1.71, *p* = 0.183] or SWLS [*F*(2,189) = 0.397, *p* = 0.673]. We found no differences; hence, the three groups remained equal except the arousal manipulation.

**FIGURE 1:**
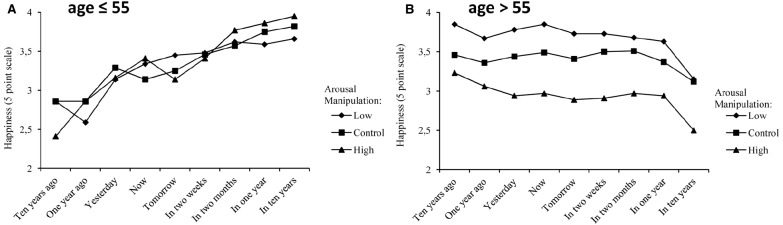
**(A)** Mean level of happiness plotted over time for participants younger than 55 years (*n* = 79). **(B)** Mean level of happiness plotted over time for participants older than 55 years (*n* = 114).

**FIGURE 2:**
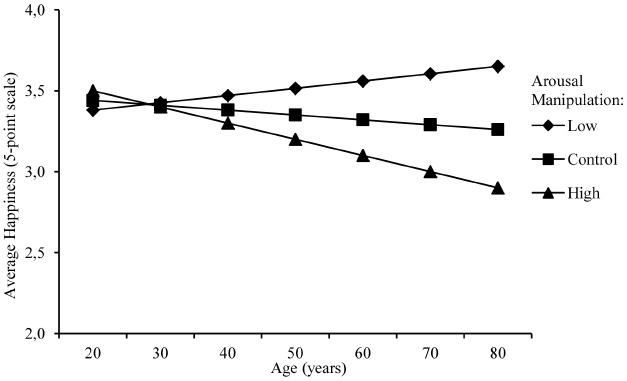
**Influence of the manipulation on average happiness based on age (*n* = 193)**.

We employed a multi level model (MLM) in R-statistics 3.0.2 using the LmerTest package for our analyses (data and R syntaxes used are available). The dependent variable is the happiness rated for a specific time, as all participants rated their happiness at nine time points (the nine time points representing if it was happiness 10 years ago, 1 year ago, yesterday, now, tomorrow, in 2 weeks, in 2 months, in 1 year, in 10 years), a repeated measures method is used. The time variable is included as a predictor and the participants have random slopes and intercepts on this variable. In addition, age (z-scored) and the arousal framing manipulation conditions (control = 0, low arousal –1, and high arousal 1) are included in the model as fixed predictors. This means that the model is centered at a participant of mean age, rating happiness 10 years ago, receiving the control condition. Figures 1 and 2 depict how participants younger and older than 55 perceived happiness across time periods. However, Figures 1A,B are visualizations of mean age differences (median split); all analyses were made with age as a continuous variable.

The MLM revealed that the arousal manipulation significantly influenced the ratings of happiness [*B* = –0.22, *t*(189) = –3.59, *p* < 0.001], 95% CI (–0.35,–0.10). The overall negative influence of arousal on happiness indicates that when described as high in arousal the overall (average of all nine time points, for all participants) ratings of happiness were lower than the control and low arousal manipulation (Figure 2). More importantly, age interacted with the arousal manipulation [*B* = –0.15, *t*(189) = –2.52, *p* = 0.013], 95% CI (–0.15,–0.28), which indicates that older adults were more influenced by the manipulation than younger adults. This can be seen by the clearly differentiated lines in Figure 1B and the influence of the manipulation on the average happiness over all nine time points, as seen in Figure 2. This shows that happiness, when primed as low in arousal, is rated as higher by older adults and when defined as high in arousal, is rated as lower by older adults. Younger adults seem to be unaffected by the manipulation, and additionally the difference is smallest for people in their 20′s and largest for people in their 80′s. No age differences were seen in the control condition (Figure 2).

In addition, we found that age had a positive influence on happiness [*B* = 0.31, *t*(190) = 5.15, *p* < 0.001], 95% CI (0.31,0.20), indicating that older adults rated their happiness as higher compared to younger (over all time points and manipulations). The analyses also showed that overall the rating of happiness increased with time [*B* = 0.03, *t*(187) = 3.44, *p* < 0.001], 95% CI (0.03,0.01), from 10 years ago, to 10 years in the future which indicates that participants perceived more happiness in their future than in their past (see Figures 1A,B). However, the negative interaction between time and age [*B* = –0.08, *t*(188) = 10.41, *p* < 0.001], 95% CI (–0.09,–0.11) indicates a more positive perception of future happiness among younger adults when compared to older adults (this can be seen by comparing slopes between Figures 1A,B).

## Discussion

Our main finding was that older adults rated happiness higher when framed as low in arousal, compared to happiness framed as high in arousal. The younger participants remained uninfluenced by the happiness framing manipulation. In addition, the manipulation seemed to influence ratings of future happiness and ratings past happiness in the same way as ratings of current happiness. These results have several important implications.

First, our results are consistent with the literature suggesting that older adults tend to avoid high arousal emotions (Pinquart, 2001; Kessler and Staudinger, 2009; Mogilner et al., 2011; English and Carstensen, 2014). And that older adults have a clear preference for low arousal positive affect over high arousal positive affect we asked to value their experiences (Scheibe et al., 2013).

Second, the fact that a manipulation (changing only a few words) of how happiness is defined (in terms of low or high arousal) systematically changed happiness ratings of both past, current, and future happiness, has important implications for future research. Our arousal framing manipulation produced age differences in happiness ratings, indicating that older adults relate more to happiness as a low arousal emotion and are consequently less happy with high arousal. Thus, our results are of general importance for research on age-related differences in emotions that rely on self-reported emotions (Scheibe and Carstensen, 2010). This could prove to be troublesome as affective measures are used as global indicators of well-being and health (Diener et al., 2010), while the mean age of citizens varies in different countries. To avoid misinterpretations of possible age effects both high and low arousal positive affect needs to be measured. In addition, comparisons and meta-studies that mix studies measuring cognitive well-being (low in arousal) and affective well-being (high in arousal) could be troublesome. Our finding suggests that high arousal measures would decline with age and low arousal measures should increase with age. This could be one explanation to the puzzle of the relationship between age and happiness (Frijters and Beatton, 2012).

Our finding fits the literature that suggests that older adults avoid high arousal emotions. Nevertheless, it could be argued that our manipulation manipulated other aspects of happiness than arousal (i.e., intensity). In addition, we cannot know if the difference represents a change in affect (so that older adults feel more satisfaction) or a change in rating of affect (so that older adults only rate their affect higher when described as satisfaction). Still, the fact that the wording of happiness and description of happiness-related conditions influence the ratings of happiness differently in younger and older adults, and produced a significant age difference, remains.

Future research should investigate possible mediators and moderators of this arousal avoidance. It could be that it is not chronological age, but rather psychological age, health, or cognitive change that is the underlying factor. Carstensen (2006) suggests that as people come closer to the end of life, psychological age changes, which leads to a motivational shift whereby people stop seeking excitement and start caring more about other aspects of life, such as relationships with people close to them. This motivational shift could be an explanation for our finding. Research has also suggested that affective and emotional processing differences are related to cognitive functioning (Mather and Carstensen, 2005; Mather and Knight, 2005), and that distraction of cognitive abilities can reverse the positivity bias in older adults (Knight et al., 2007). This would indicate that cognitive decline in older adults is the underlying factor to emotional and arousal avoidance. Further study is needed to examine why older adults prefer low over high arousal happiness.

As a final note, further longitudinal research can answer if this finding is connected to aging effects or cohort effects, however, today’s researchers of happiness need to consider that the wording of happiness and happiness-related questions can have different influences on younger and older adults. Thus, researchers need to measures affective states both high and low in arousal to be able to draw conclusions on age-related changes in affect.

In sum, our study shows that a change in wording of items can accentuate age differences. From a theoretical standpoint, we demonstrate that both valence and arousal determine preference of type of affect (Russell, 2003), and that older adults rate their happiness as higher when happiness is framed as low in arousal.

### Conflict of Interest Statement

The authors declare that the research was conducted in the absence of any commercial or financial relationships that could be construed as a potential conflict of interest.
